# The relationship between openness to experience and college students’ well-being: the mediating roles of art-related aesthetic perception and needs

**DOI:** 10.3389/fpsyg.2026.1834660

**Published:** 2026-06-15

**Authors:** Yi Jin, Xiaohang Wang, Xingfa Long

**Affiliations:** 1 School of Humanities and Museology (Art Research Institute), Nanjing University of the Arts, Nanjing, China; 2 Center for Mental Health and Psychological Studies, Hohai University, Nanjing, China; 3 Department of Human Development and Family Studies, Faculty of Human Ecology, Universiti Putra Malaysia, Serdang, Malaysia; 4 Quzhou College of Technology, Quzhou, China

**Keywords:** art-related aesthetic needs, art-related aesthetic perception, openness to experience, psychological well-being, subjective well-being

## Abstract

**Objectives:**

Grounded in Self-Determination Theory, this study investigated the impact of openness to experience on both subjective and psychological well-being among college students, with a specific focus on the mediating mechanisms of art-related aesthetic perception and needs.

**Methods:**

A sample of 558 college students (*M_age_* = 18.40, *SD* = 1.38) completed standardized assessments. Mediation modeling was employed to test the direct and indirect pathways between personality traits and well-being.

**Results:**

Openness to experience did not directly predict either dimension of well-being. Instead, it showed indirect associations with well-being through two pathways. (1) The independent mediation of art-related aesthetic needs, and (2) a sequential mediation involving both art-related aesthetic perception and needs.

**Conclusion:**

These findings clarify the psychological mechanisms by which aesthetic traits foster well-being, highlighting the critical role of art-related aesthetic needs in translating personality into positive adjustment. While providing cross-sectional evidence, future research should utilize longitudinal designs to confirm causal directions.

## 1 Introduction

Well-being is a vital psychological construct for assessing an individual’s quality of life and mental health status (Yan and Dong, 2020; Guo, 2019). Although some researchers have categorized well-being into multiple dimensions, such as social, subjective well-being, and psychological well-being, most psychologists tend to conceptualize it as a subjective experience or psychological state resulting from the fulfillment of one’s needs (Yan and Zheng, 2006). In recent years, the well-being of college students and its influencing factors have become prominent topics in psychological research, receiving growing academic attention (Gao and Zhang, 2015).

Two major paradigms have dominated the study of well-being: *Psychological Well-Being* (PWB) and *Subjective Well-Being* (SWB). These frameworks, respectively, explore the essence of happiness from the perspectives of internal psychological functioning and subjective life experiences. PWB originates from humanistic and positive psychology and emphasizes the full development and realization of individual potential. Ryff’s (1989) model conceptualizes PWB as a dynamic evaluation of self-actualization, encompassing six key dimensions: autonomy, environmental mastery, personal growth, positive relations with others, purpose in life, and self-acceptance. Unlike momentary emotional experiences, PWB reflects a relatively enduring and comprehensive evaluation of one’s functioning and self-realization, encompassing multiple dimensions such as autonomy, personal growth, and purpose in life. Philosophically, it aligns with Aristotle’s concept of *Eudaimonia*, which views happiness as human flourishing achieved through virtuous living and purposeful engagement.

In contrast, subjective well-being (SWB) centers on individuals’ subjective evaluations of their lives, comprising two core components: affective experiences (i.e., the balance between positive and negative affect) and cognitive appraisal (i.e., life satisfaction). As defined by Diener (1984), SWB represents an individual’s global subjective evaluation of life quality. Although SWB is sensitive to situational and temporal influences, prior research suggests that it also demonstrates a degree of stability over time, reflecting both state and trait characteristics (Diener et al., 2018). This stability is evident in the relative consistency of individuals’ life satisfaction and affective tendencies, rather than being limited to transient emotional experiences.

Joshanloo (2016) noted that although subjective well-being (SWB) and psychological well-being (PWB) are theoretically related, they may differ in how individuals experience well-being in practice. This perspective is further supported by Keyes (2007), who suggested that SWB and PWB reflect two complementary traditions in well-being research, with differences in conceptual emphasis, research focus, and experiential aspects, while remaining closely related rather than entirely independent constructs.

As two different research orientations within the well-being literature, SWB is generally understood as an individual’s subjective evaluation of life quality based on personal standards, such as the enjoyment derived from everyday activities and overall life satisfaction. In this sense, SWB is often described as a relatively spontaneous form of well-being that does not necessarily require sustained self-regulation or deliberate effort (Vittersø and Nilsen, 2002). Therefore, scholars such as Diener, Suh, and Lucas conceptualize subjective well-being as reflecting happiness, characterized by high life satisfaction, more frequent experiences of positive emotions, and correspondingly fewer negative emotions in daily life.

PWB refers to a state of optimal psychological functioning, achieved through the full realization of one’s potential and values, leading to self-perfection, self-actualization, and personal accomplishment (Ryff, 1995). Ryff contended that simple happiness does not equate to well-being; rather, well-being arises when individuals express their potential to the fullest through deeply engaging activities. Therefore, unlike subjective well-being, which focuses on the experience of simple pleasures in life, psychological well-being places greater emphasis on the realization of self-potential and self-worth. The pursuit of PWB typically involves longer time-frames, greater challenges, and resilience in the face of setbacks, ultimately reflecting the fulfillment gained through self-actualization.

Based on prior research, subjective well-being (SWB) and psychological well-being (PWB) can be understood as related yet distinguishable aspects of well-being, which may differ in individuals’ experiential emphasis. Therefore, the relationship between SWB and PWB can be characterized as complementary, with meaningful distinctions rather than clear-cut separations. Importantly, rather than representing opposing or hierarchical forms of well-being, SWB and PWB capture complementary dimensions of human functioning.

Previous research has identified a range of factors that affect well-being among college students, including attachment style (Zhang et al., 2022), parenting style (Stavrulaki et al., 2021), childhood maltreatment (Bartolomé-Valenzuela et al., 2024), social media use (Saleem and Hawamdeh, 2023), and personality traits (Yu et al., 2024; Karris Bachik et al., 2021; Fino and Sun, 2022; Yin and Li, 2015). Among these, personality has emerged as a key predictor of well-being. Diener et al. (2018) research group pointed out that personality acts as a powerful predictor of well-being. A longitudinal study tracking 360 college students over 1 year found that well-being may function as a relatively stable personality trait (Yin and Li, 2015). It is theoretically and practically meaningful to identify personality traits associated with well-being, as such efforts contribute to the enhancement of individual happiness and emotional resilience. However, the majority of prior research has rarely considered subjective well-being and psychological well-being concurrently (Disabato et al., 2016). Accumulated evidence indicates that SWB and PWB represent distinct but interrelated constructs, which together characterize positive human functioning (Garcia et al., 2017; Keyes, 2002). For this reason, simultaneous investigation of both dimensions is warranted. Building on this theoretical foundation, the present study seeks to examine the associations between personality and the dual dimensions of well-being.

### 1.1 Openness to experience and students’ well-being

The relationship between the Big Five personality traits and well-being has been extensively studied across both Western and Eastern cultural contexts (Steel et al., 2008; Zhang et al., 2020). Research consistently shows that openness to experience, conscientiousness, extraversion, and agreeableness are positively associated with well-being, whereas neuroticism is negatively associated (Anglim et al., 2020). Openness to experience is characterized by imagination, creativity, intellectual curiosity, and aesthetic sensitivity (McCrae, 1987). McCrae and Sutin (2009) further demonstrated that individuals high in openness tend to possess greater intellectual curiosity and aesthetic sensitivity. Such individuals actively seek out new experiences, maintain openness toward external stimuli, and show a willingness to establish positive social connections. These behaviors often contribute to a stronger sense of meaning in life and enhanced self-congruence, which resonates with the autonomy need highlighted in self-determination theory. Within the framework of self-determination theory, Deci and Vansteenkiste (2004) proposed that every individual possesses an inherent and innate tendency toward self-actualization, personal growth, and continual self-integration. When individuals engage in more autonomous rather than controlled behaviors, their need for autonomy is fulfilled, thereby maintaining higher levels of well-being. Supporting this, Sheldon et al. (1997) found that individuals with stronger social connectedness and greater tendencies for emotional self-disclosure tend to report higher levels of well-being. Collectively, these findings suggest that openness to experience aligns closely with the autonomy need emphasized in self-determination theory and contributes to well-being through the enhancement of self-congruence. In addition to personality traits, domain-specific experiences such as art-related aesthetic engagement may also contribute to the satisfaction of basic psychological needs.

Moreover, openness is associated with higher levels of art-related aesthetic sensitivity and interest in novel experiences, which enables open individuals to experience positive art-related aesthetic emotions and profound affective responses (e.g., awe, curiosity, being moved) during artistic encounters. These affective responses, in turn, can enhance well-being (Cotter et al., 2024). Cotter et al. (2024) also found that, among the Big Five traits, openness demonstrated the strongest correlation with immersion, a state positively associated with positive affect and negatively associated with negative affect, and predictive of increases in well-being over time. Their findings suggest that immersion is a key psychological mechanism linking openness to well-being.

Building on this evidence, the present study focuses on the role of openness to experience within the Big Five in shaping well-being. Accordingly, we propose the first hypothesis:

*Hypothesis 1*: Openness to experience directly predicts well-being.

### 1.2 The mediating role of art-related aesthetic perception and needs

The art-related aesthetic perception refers to an individual’s capacity to respond to aesthetic stimuli, emphasizing a trait-like disposition. Individuals high in aesthetic responsiveness are more likely to experience aesthetic cognitions, emotions, and related physiological reactions more frequently, and are also more inclined to engage in artistic activities (Schlotz et al., 2021). In contrast, art-related aesthetic needs place greater emphasis on the active perception of beauty and excellence, deep appreciation, and emotional responses, particularly involving the sustained pursuit and experience of artistic and aesthetic values (Güsewell, 2013). In this study, art-related aesthetic needs are conceptualized as an individual’s artistic needs, reflecting the tendency to actively seek, engage with, and derive fulfillment and meaning from artistic activities. AReA highlights the ability and dispositional tendency to “perceive and respond,” whereas art-related aesthetic needs reflect the aspiration–pursuit–fulfillment characteristics of an individual’s demand for art.

Self-Determination Theory (SDT) posits that human beings have three basic psychological needs: autonomy, competence, and relatedness. The satisfaction of these needs is essential for maintaining psychological integrity and promoting well-being across the lifespan, and has been consistently associated with a range of positive outcomes, including better physical health (Ng et al., 2012) and higher levels of well-being (Chen et al., 2014).

Although aesthetic needs are not explicitly identified as basic needs within SDT, art-related aesthetic experiences may serve as important experiential pathways through which these needs are fulfilled. Previous research has also suggested that engagement in aesthetic or artistic activities may contribute to the satisfaction of basic psychological needs (Zeng and Guo, 2012; Cotter et al., 2024). Specifically, engagement with artistic activities often involves self-directed exploration and intrinsic interest, thereby supporting autonomy; the process of perceiving and interpreting artistic forms may foster a sense of competence; and shared or emotionally resonant aesthetic experiences can enhance feelings of relatedness.

From this perspective, art-related aesthetic perception and needs can be understood as domain-specific processes that contribute to the satisfaction of basic psychological needs, and thereby promote well-being.

In traditional Chinese culture, well-being has not been regarded as a mere subjective feeling, nor simply as “happiness” (le) or “fortune” (fu). Rather, it has been closely associated with goodness (shan) and beauty (mei). The appreciation of beauty, such as natural scenery, poetry, and art, has long been considered an important source of subjective well-being (Zeng and Guo, 2012). Openness to experience is strongly linked to such aesthetic sensitivity and depth of perception, which can further enrich an individual’s experience of well-being (Martínez-Martí et al., 2016). Art related aesthetic activities, by fostering creativity and self-expression, also promote self-actualization, a core component of PWB. For example, in art therapy, self-integration through expressive media such as drawing can foster psychological healing and self-acceptance.

Art-related aesthetic experiences, such as viewing art, can elicit immediate positive affect and activate dopaminergic reward pathways, thereby enhancing SWB. Brajša-Žganec et al. (2011) argued that material wealth is neither a necessary nor sufficient condition for well-being. Oishi et al. (1999) further noted that once basic needs are met, cultural factors, like aesthetic values, play a more prominent role in shaping well-being. Art, by facilitating temporary transcendence from daily struggles and enabling symbolic expression of internal conflicts, can serve as a potent well-being enhancer.

Specifically, drawing allows individuals to project and process repressed emotions, motivations, and internal conflicts in a safe and symbolic medium. Because drawings are often emotionally neutral and symbolic, they enable deeper psychological exploration with reduced defensiveness (Malchiodi, 2003). McNiff (2020) suggested that aesthetic creation itself functions as a cognitive regulatory mechanism, while Hogan (2001) emphasized the therapeutic and integrative role of art in linking affective expression and cognitive processing.

Art-related esthetic self-expression fosters emotional catharsis, enhances self-awareness, and promotes cognitive restructuring and self-acceptance. For example, drawing provides a psychologically safe space in which participants can explore their internal world, restructure self-perceptions, and build capacities for self-reflection and emotional healing, all of which contribute to greater well-being.

Aesthetics is central to art, and the psychological benefits of aesthetic activity are fundamentally rooted in the active pursuit of aesthetic experience. According to Self-Determination Theory, well-being increases when basic psychological needs are met. Art-related aesthetic experiences can satisfy the needs for autonomy (freely engaging in art-related aesthetic choices), competence (understanding and appreciating art), and relatedness (connecting with others through shared aesthetic appreciation). Thus, fulfilling art-related aesthetic needs not only leads to immediate pleasure but also contributes to long-term increases in both PWB and SWB.

Based on the above theoretical framework, art-related aesthetic perception and needs can be understood as domain-specific pathways through which openness to experience is associated with well-being. Specifically, openness to experience enhances individuals’ sensitivity to aesthetic stimuli, thereby facilitating richer art-related aesthetic perception. This heightened perception may, in turn, be internalized into stronger aesthetic needs, reflecting a motivational drive to seek, engage in, and derive meaning from aesthetic experiences. Through this “perception – need” pathway, openness to experience is indirectly translated into both subjective and psychological well-being. Specifically, we hypothesize:

*Hypothesis 2*: Openness to experience will indirectly predict well-being through art-related aesthetic perception.

*Hypothesis 3*: Openness to experience will indirectly predict well-being through art-related aesthetic needs.

*Hypothesis 4*: There will be a sequential mediating effect, whereby openness to experience predicts greater art-related aesthetic perception, which in turn enhances art-related aesthetic needs, ultimately promoting well-being.

### 1.3 The current study

**Figure 1:**
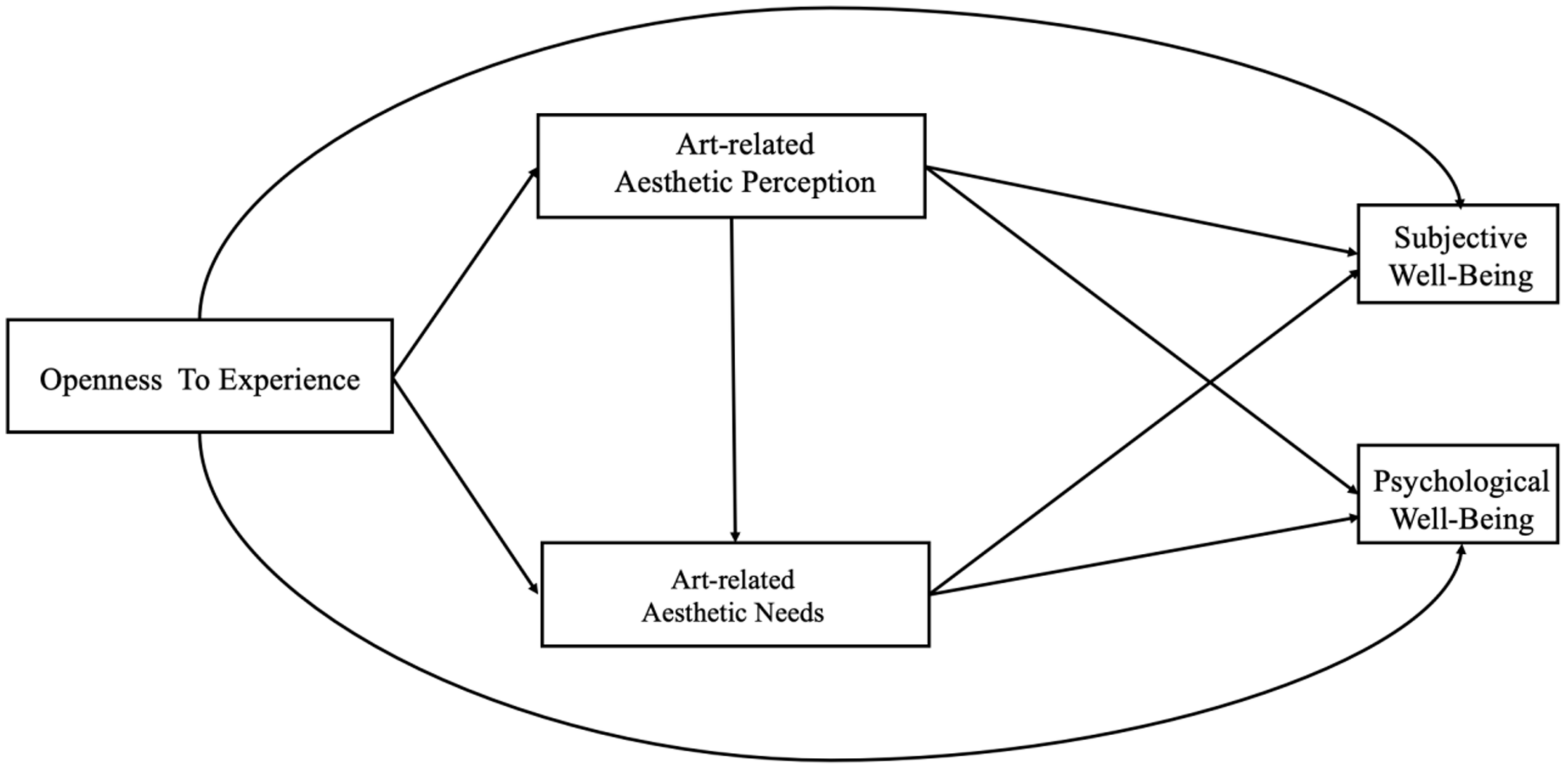
An indirect effects model linking openness to experience with subjective and psychological well-being through art-related aesthetic perception and needs. PWB is psychological well-being; SWB is subjective well-being.

While prior literature has predominantly established the link between openness to experience, behavioral art engagement, and well-being (Cotter et al., 2024), the internal psychological precursors that drive this behavioral engagement remain largely unpacked. Existing studies often treat art appreciation or engagement as a holistic behavioral construct. The current study departs from this by disentangling the internal psychological mechanisms into two distinct constructs: art-related aesthetic perception (the cognitive capacity and sensitivity to detect and process aesthetic qualities) and art-related aesthetic needs (the intrinsic motivational drive to seek aesthetic fulfillment). By distinguishing these constructs, we identify a novel sequential mechanism extending the Self-Determination Theory (SDT). We posit that cognitive perception alone is insufficient for sustained well-being; rather, perception should be internalized and transformed into a psychological need. This ‘perception–need–fulfillment’ pathway elucidates how personality traits shape well-being not just through outward engagement, but by cultivating profound internal aesthetic drives. Furthermore, this study explores the differential pathways through which these internal aesthetic mechanisms are associated with subjective well-being (SWB) versus psychological well-being (PWB). We propose an indirect effect model (see Figure 1) in which openness to experience is associated with well-being via its relations with art-related aesthetic perception and needs.

## 2 Method

### 2.1 Procedure and participants

The present study was conducted in October 2024. Five universities in Jiangsu Province, China, were randomly selected, and a convenience sampling method was used to recruit college students for an online survey (mean age = 18.40 ± 1.38, age range = 18–26 years). The questionnaire was distributed via the “Wenjuanxing” program, which was linked to the WeChat platform for convenience sampling (Jin et al., 2022; Wu et al., 2020). WeChat is one of the most widely used communication applications in China, with over 1.2 billion monthly active users (Jin et al., 2022). Participants were required to meet the following criteria: having the ability to read and write in Chinese, completing the questionnaire independently, and participating voluntarily. Prior to the survey, all participants signed an electronic informed consent form.

To improve data validity, questionnaires completed in less than 3 min were excluded, along with those with more than one-third missing responses, patterned or inconsistent responses, or obvious errors. After screening, 14 invalid responses were removed, leaving 558 valid questionnaires, yielding a valid response rate of 97.55%. The demographic characteristics of the participants are presented in Table 1.

**Table 1:** Demographic characteristics of the participants (*N* = 558).

Variables	*N*	Percent (%)
Gender		
Male	312	55.91%
Female	246	44.09%
Art aesthetic experiences or engagements		
Yes	416	74.55%
No	142	25.45%
Ongoing aesthetic activities		
Music, dance	131	23.48%
Painting, writing	107	19.18%
Other arts	92	16.49%
None	228	40.85%

### 2.2 Measures

#### 2.2.1 Openness to experience

Openness to experience was measured using a shortened version of the NEO Personality Inventory-Revised (*NEO-PI-R*) adapted by Hsu et al. (2011), which consists of 15 items in total. Three items were selected to assess openness to experience: (1) “*I am very interested in the arts, such as music and visual arts*”; (2) “*Sometimes, when reading poetry or looking at a piece of art, I feel a deep emotional stirring*”; and (3) “*I enjoy encountering new experiences and learning new things.*” All items were rated on a 4-point Likert scale, ranging from 1 (*strongly disagree*) to 4 (*strongly agree*), with higher scores indicating greater openness to experience. In the present study, the Cronbach’s alpha coefficient for openness to experience was 0.73.

#### 2.2.2 Art-related aesthetic perception

Art-related aesthetic perception was assessed using the Chinese version of the Aesthetic Responsiveness Assessment (AReA) revised by based on the cultural context of China. The original AReA was developed by Schlotz et al. (2021) and has demonstrated good psychometric properties. The scale comprises three subscales: aesthetic appreciation (5 items; e.g., “*I notice the beauty when appreciating works of art*”), intense aesthetic experience (4 items; e.g., “*When appreciating art, I sometimes experience feelings of awe, fear, or profound emotional impact*”), and creative behavior (3 items; e.g., “*Engaging in sculpture, painting, directing films, or working in design*”). All items were rated on a 5-point Likert scale, from 1 (*never*) to 5 (*always*), with higher scores indicating a higher level of art-related aesthetic perception. In the current study, the Cronbach’s alpha coefficient for art-related aesthetic perception was 0.93. The reliability coefficients for each dimension were also satisfactory: aesthetic appreciation Cronbach’s alpha = 0.87, intense aesthetic experience Cronbach’s alpha = 0.76, and creative behavior Cronbach’s alpha = 0.87.

#### 2.2.3 Art-related aesthetic needs

Art-related aesthetic needs was measured using the Appreciation of Beauty and Excellence subscale from the Values in Action Inventory of Strengths (VIA-IS-ABE), originally developed by Peterson et al. (2008) and revised for the Chinese cultural context by Duan et al. (2011). The subscale includes 10 items (e.g., “*I often long for experience great works of art, such as music, drama, or painting*” and “*I greatly enjoy* var*ious forms of art*”). All items were rated on a 5-point Likert scale, ranging from 1 (*strongly disagree*) to 5 (*strongly agree*). Higher scores reflect a greater need for art. In this study, the Cronbach’s alpha coefficient for need for art was 0.92.

#### 2.2.4 Psychological well-being

Psychological well-being was defined as the level of happiness experienced based on individuals’ subjective evaluations of their lives (Diener and Ryan, 2009). It was measured using a 10-item scale adapted from Blais et al. (1999). Participants rated each item on a 7-point Likert scale, ranging from 1 (*strongly disagree*) to 7 (*strongly agree*), with higher scores indicating higher levels of psychological well-being (e.g.*, “considering my current physical condition, I am content with what I am capable of accomplishing” and “I am confident in my ability to maintain important relationships”*). In the present study, the Cronbach’s alpha coefficient for psychological well-being was 0.94.

#### 2.2.5 Subjective well-being

Subjective well-being was assessed using the Subjective Well-Being Scale developed by Diener et al. (2000) and later revised for the Chinese context by Yan and Zheng (2006). The scale consists of 19 items across three dimensions: positive affect, negative affect, and life satisfaction. Items measuring positive and negative affect were rated on a 7-point scale, with 1 indicating “not at all” and 7 indicating “all the time,” with the negative affect dimension reverse-scored. Life satisfaction items were rated from 1 (*strongly disagree*) to 7 (*strongly agree*). Higher scores reflect greater levels of subjective well-being (e.g.*, “most aspects of my life are close to my ideal” and “so far, I have gotten most of the things I want in life”*). In this study, the Cronbach’s alpha coefficient for subjective well-being was 0.91.

### 2.3 Data analysis

All data analyses were conducted using R software version 4.2.3. Descriptive statistics and correlation analyses were first performed for the collected variables. Finally, the indirect effect model examining the effect of openness to experience on well-being among university students was analyzed using the ‘lavaan’ package in R.

## 3 Results

### 3.1 Common method bias test

All variables in this study were measured via participants’ self-reports, which may introduce common method bias (Kock et al., 2021). Harman’s single-factor test was conducted to examine common method bias. The results indicated that exploratory factor analysis extracted 11 factors with eigenvalues greater than 1. The first factor accounted for 20.73% of the total variance, which is below the 40% critical threshold. Therefore, severe common method bias was not present in this study.

### 3.2 Preliminary analysis

Descriptive statistics for the variables under study, including means, standard deviations, and zero-order correlations, are shown in Table 1, as anticipated. The correlation analysis results indicated that openness to experience, art related aesthetic perception, art-related aesthetic needs, psychological well-being, subjective well-being among university students were all positively correlated with each other (*p*s < 0.001). Detailed correlation data can be found in Table 2.

**Table 2:** Descriptive statistics and correlations of the main study variables.

Variables	*M*	*SD*	1	2	3	4	5
1. Openness to experience	3.06	0.76	–				
2. Art-related aesthetic perception	3.58	0.76	0.68^***^	–			
3. Art-related aesthetic needs	3.99	0.73	0.64^***^	0.71^***^	–		
4. Psychological well-being	5.38	1.11	0.35^***^	0.38^***^	0.49^***^	–	
5. Subjective well-being	4.70	0.90	0.28^***^	0.34^***^	0.39^***^	0.81^***^	–

^***^p < 0.001.

### 3.3 Testing for indirect effect

**Figure 2:**
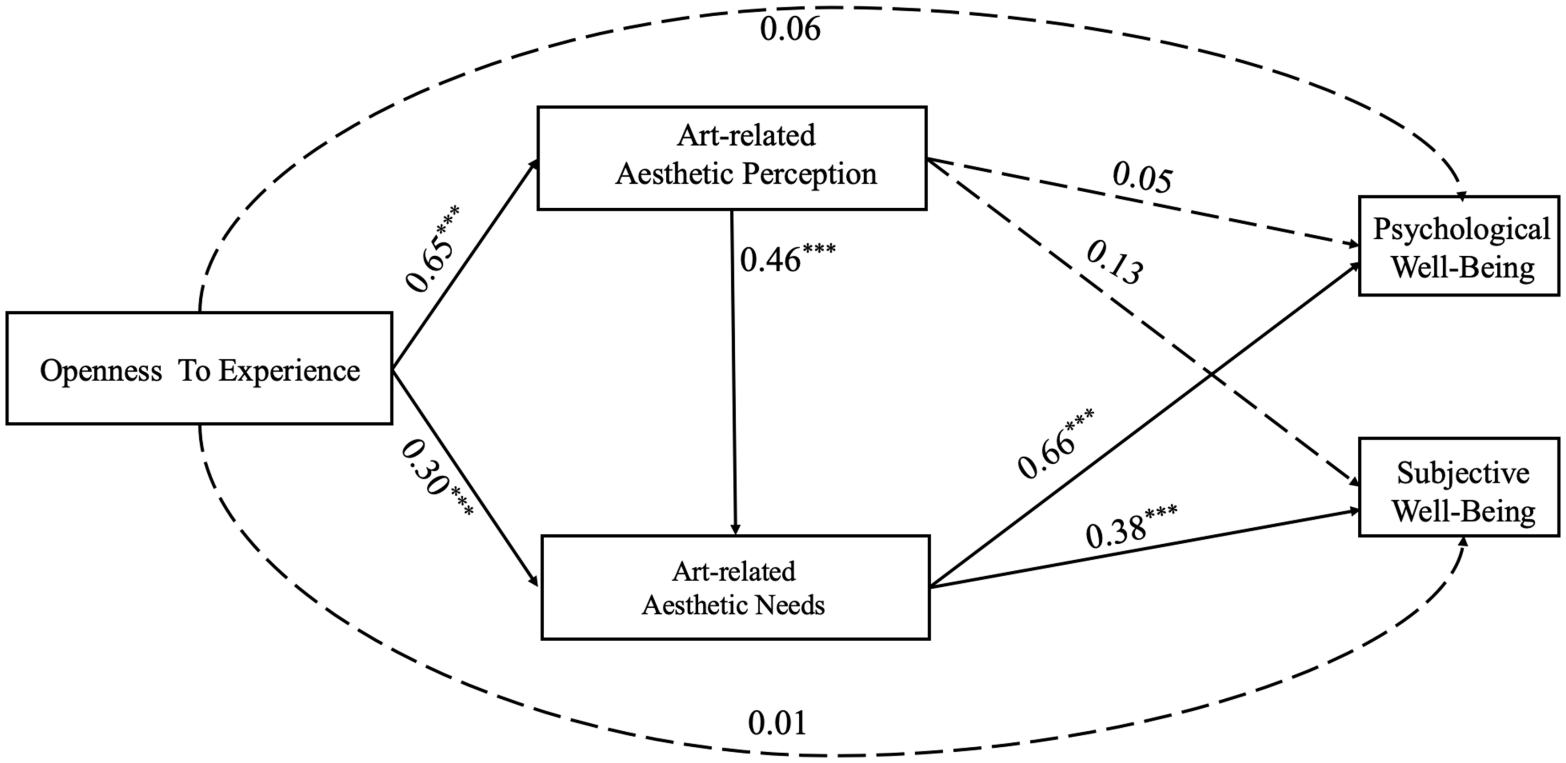
Indirect effect model of well-being among university students. ^***^p < 0.001.

A structural equation model was used to explore the mechanisms through which art-related aesthetic perception and art-related aesthetic needs mediate the effects of openness to experience on psychological and subjective well-being among university students (Figure 2). The model demonstrated a good fit: *χ^2^/df* = 3.946, *CFI* = 0.978, *TLI* = 0.952, *GFI* = 0.970, *RMSEA* = 0.073, *SRMR* = 0.034. The significance of the indirect effect was tested using the bootstrap method (with 5,000 resamples), and the results showed the following: (1) Openness to experience had no significant direct effect on psychological well-being (*β* = 0.06, *p* = 0.418 > 0.05, *95% CI* = [−0.086, 0.199]) and subjective well-being (*β* = 0.01, *p* = 0.849 > 0.05, *95% CI* = [−0.114, 0.134]); (2) Openness to experience had a significant indirect effect on psychological well-being (*β* = 0.20, *p* < 0.001, *95% CI* = [0.113, 0.293]) and subjective well-being (*β* = 0.11, *p* < 0.001, *95% CI* = [0.066, 0.172]) through art-related aesthetic needs; (3) Openness to experience had a significant indirect effect on psychological well-being (*β* = 0.20, *p* < 0.001, *95% CI* = [0.129, 0.271]) and subjective well-being (*β* = 0.11, *p* < 0.001, *95% CI* = [0.066, 0.165]) via art related aesthetic perception and needs. Overall, openness to experience did not directly affect psychological and subjective well-being among university students, but rather were associated with both through art-related aesthetic perception and needs. Detailed results are presented in Table 3.

**Table 3:** Effect size analysis of the indirect effect paths of art-related aesthetic perception and needs on well-being.

Pathways	Standard effects	Boot S. E	95%CI	
			LLCI	ULCI
Openness to experience → PWB	0.06	0.07	−0.086	0.199
Openness to experience → SWB	0.01	0.06	−0.114	0.134
Openness to experience → art-related aesthetic perception → PWB	0.04	0.05	−0.066	0.148
Openness to experience → art-related aesthetic perception → SWB	0.08	0.05	−0.008	0.174
Openness to experience → art-related aesthetic needs → PWB	**0.20**	**0.05**	**0.113**	**0.293**
Openness to experience → art-related aesthetic needs → SWB	**0.11**	**0.03**	**0.066**	**0.172**
Openness to experience → art-related aesthetic needs → PWB	**0.20**	**0.03**	**0.129**	**0.271**
Openness to experience → art-related aesthetic perception → art-related aesthetic needs → SWB	**0.11**	**0.03**	**0.066**	**0.165**

PWB is psychological well-being; SWB is subjective well-being. Black bold lines indicate statistically significant paths in the figure.

To further validate the stability of the observed mediation pathways, we compared our primary model (see Figure 2) with a higher-order model (see Supplementary material) where subjective and psychological well-being were integrated into a single latent construct of “Well-Being.” The results from this higher-order model remained consistent with the separate models: openness to experience was related to well-being only through the independent mediation of art-related aesthetic needs and the sequential mediation of aesthetic perception and needs. Notably, while the pathways were consistent, the effect sizes were more pronounced when modeling PWB and SWB separately (e.g., *β_PWB_* = 0.20 vs. *β_wb_* = 0.08). This suggests that although the mechanism is robust, separating the two dimensions of well-being provides greater statistical sensitivity and captures the nuanced impact of aesthetic traits on different facets of adolescent adjustment.

## 4 Discussion

### 4.1 The role of openness in college students’ well-being

This study examined the relationship between openness to experience, one of the Big Five personality traits, and college students’ subjective well-being and psychological well-being, as well as the mediating roles of art-related aesthetic perception and needs in this relationship. This study extends the application of SDT by identifying art-related aesthetic needs as a domain-specific motivational pathway through which openness is associated with the fulfillment of basic psychological needs. While SDT traditionally emphasizes the fulfillment of autonomy, competence, and relatedness as the core determinants of well-being, the current findings suggest that art-related aesthetic needs may also play a vital role in satisfying individuals’ intrinsic psychological requirements. Specifically, openness to experience enhances art-related aesthetic perception, which subsequently stimulates art-related aesthetic needs and promotes both subjective and psychological well-being. This sequential mechanism provides empirical support for the SDT assumption that personality traits are indirectly related to well-being through the satisfaction of internalized needs (Wang et al., 2019).

Moreover, by highlighting the role of art-related aesthetic perception as a motivational trigger that elicits intrinsic satisfaction, the study enriches SDT’s account of how intrinsic motivation arises and sustains well-being. Collectively, these findings broaden the theoretical scope of SDT by integrating aesthetic experiences into the framework of need satisfaction and intrinsic motivation.

However, the results of the present study indicate that openness to experience does not significantly and positively predict college students’ well-being. This suggests that openness, in itself, is not a direct determinant of subjective well-being or psychological well-being. Within the Chinese cultural context, the construct of openness shares commonalities with Western individualistic cultures while also reflecting culture-specific characteristics (Cheung et al., 2008). It encompasses not only cognitive curiosity and exploratory tendencies but also elements of social and interpersonal meaning. Traditional Chinese culture places great emphasis on the appreciation of beauty, such as natural landscapes, poetry, and art, which has been regarded as an important pathway to enhancing subjective well-being (Zeng and Guo, 2012). Openness has been found to be strongly associated with the appreciation of beauty (Martínez-Martí et al., 2016), which may help explain its correlation with life satisfaction. Nevertheless, this trait primarily emphasizes cognitive enrichment, curiosity, and receptivity to new experiences rather than directly involving psychological resources more closely tied to well-being, such as emotion regulation or social support (Kim et al., 2016). As a result, the cognitive exploration and aesthetic tendencies associated with openness may not necessarily or consistently translate into positive emotional experiences, thereby limiting its direct predictive role in well-being.

### 4.2 Indirect effect of art-related aesthetic needs

Interestingly, the study identified a full indirect effect of art-related aesthetic needs in the relationship between openness to experience and both subjective well-being and psychological well-being. This finding indicates that openness to experience may enhance well-being indirectly by increasing individuals’ art-related aesthetic needs. Consistent with previous literature, openness to experience has been repeatedly associated with increased aesthetic sensitivity and greater engagement in aesthetic activities (Swami and Furnham, 2020; Kelleher-Clarke et al., 2023). Individuals with high levels of openness to experience are more likely to participate in and appreciate the arts, and such engagement has been associated with higher levels of well-being (Cotter et al., 2024). When art-related aesthetic needs are fulfilled, they can generate positive and constructive forces within the individual. An inner world imbued with kindness and beauty allows one to be more deeply moved by the goodness inherent in things through aesthetic experiences. As a developmental higher-order need, second only to self-actualization, art-related aesthetic needs provide a form of transcendent pleasure, and their fulfillment is particularly vital for individuals in the process of growth. Conversely, the deprivation of art-related aesthetic needs may lead to maladaptive states, as “the deprivation of beauty” itself has been suggested to induce illness (Jin, 2020). For instance, a study involving 890 adults found that openness was strongly associated with art-related aesthetic participation, and that such participation was linked to higher well-being compared to activities such as reading (Cotter et al., 2024). For individuals with high openness to experience, engagement in art-related aesthetic activities, such as music, painting, and literary appreciation, may not only satisfy their deeper art-related aesthetic needs and drive for exploration, but also elicit positive emotions and foster psychological resilience. These processes ultimately contribute to enhanced subjective well-being and promote both psychological and physiological health.

### 4.3 The sequential indirect effect of art-related aesthetic perception and needs

Nonetheless, a significant pathway was observed: openness to experience was associated with well-being through enhanced art-related aesthetic perception, which in turn stimulated greater art-related aesthetic needs, ultimately leading to increased well-being. On the one hand, individuals high in openness to experience tend to possess a more nuanced art-related aesthetic perception and are more likely to recognize complexity, uniqueness, and emotional depth in aesthetic experiences (Chamorro-Premuzic and Furnham, 2004; Furnham, 2021; McManus and Furnham, 2006). This heightened sensitivity fosters stronger cognitive and emotional engagement with aesthetic stimuli (Palumbo et al., 2023). As art-related aesthetic perception deepens, individuals develop stronger aesthetic motivations and needs, becoming more inclined to seek, understand, and create aesthetic experiences (Martínez-Martí et al., 2016). On the other hand, when individuals engage in meaningful and self-directed aesthetic experiences that foster a sense of competence or interpersonal resonance, their core psychological needs are satisfied, thereby enhancing positive affect and overall well-being (Deci and Ryan, 2012). When individuals derive fulfillment from aesthetic experiences, particularly in contexts where they voluntarily engage, feel competent, or experience resonance with others, their basic psychological needs of autonomy, competence, and relatedness are satisfied. This satisfaction, in turn, fosters positive emotions and psychological well-being. In other words, the fulfillment of art-related aesthetic needs is not merely a cognitive response but a process that activates and nurtures intrinsic motivation and fundamental needs.

## 5 Limitation and implication

This study has several limitations. This study has several limitations that should be acknowledged. First, the sample was drawn from university students in Jiangsu Province, which may limit the generalizability of the findings to the broader population of Chinese college students. Although participants were recruited from multiple universities, future research should include more diverse samples across different regions and developmental stages (e.g., adolescents) to enhance external validity. Second, the use of a cross-sectional design precludes causal inference. Although the proposed model was theoretically grounded, the observed relationships should be interpreted as associative rather than causal. In addition, reverse or reciprocal relationships cannot be ruled out; for instance, higher levels of well-being may also promote greater engagement in aesthetic experiences. Future studies are encouraged to adopt longitudinal, experimental, or cross-lagged designs to better examine temporal ordering and underlying mechanisms. Third, all variables were assessed using self-report measures collected at a single time point, which may introduce common method bias, shared variance, and social desirability bias. Although additional analyses suggested that common method bias was not a serious concern in the present study (see Results section), caution is still warranted. Moreover, the use of an online survey may raise concerns regarding robustness and replicability (Aguinis et al., 2021; Chmielewski and Kucker, 2020). Future research should incorporate multi-method approaches (e.g., behavioral or multi-informant data) and replicate findings across independent samples to strengthen robustness. Fourth, while the present study adopts a Self-Determination Theory (SDT) framework, future research could also benefit from integrating complementary perspectives, such as meaning-making or broader aesthetic experience theories, to provide a more comprehensive understanding of how aesthetic processes contribute to well-being. Additionally, the present study was conducted within a Chinese cultural context, where aesthetic appreciation has traditionally been emphasized as an important component of personal cultivation and well-being. As a result, participants in this study may exhibit relatively higher baseline levels of art-related aesthetic perception and needs compared to populations from other cultural backgrounds. This cultural context may be associated with both the predictors (e.g., openness to experience) and outcome variables (well-being), potentially amplifying the observed relationships. Therefore, caution should be exercised when generalizing the findings to other cultural contexts. Furthermore, the present study did not include potentially relevant control variables, such as prior artistic exposure, socioeconomic status (SES), or educational background, which may be associated with individuals’ access to aesthetic experiences and their well-being. Future research should incorporate these factors to provide a more nuanced understanding of the proposed mechanism.

Despite these limitations, the findings of this study provide robust evidence for the mediating role of art-related aesthetic needs in the relationship between openness and subjective well-being among college students. Furthermore, the study highlights a significant indirect effect pathway: openness enhances art-related aesthetic perception, which in turn stimulates higher levels of art-related aesthetic needs, thereby indirectly improving well-being. This finding not only sheds light on the mechanisms of art-related aesthetic perception and needs in fostering well-being among college students but also provides novel empirical support to the literature, reaffirming the important role of openness in promoting well-being. Beyond theoretical contributions, the findings of this study offer timely practical implications, particularly within the socio-cultural context of contemporary China. Recently, macro-level initiatives in China have placed an unprecedented emphasis on aesthetic education and cultural development as core components of youth mental health. For instance, the Ministry of Education of the People’s Republic of China (2024) launched the ‘School Aesthetic Education Immersion Action’ (Meiyu Jinrun), aiming to integrate aesthetic education across all educational stages to enhance students’ humanistic literacy and psychological resilience. Similarly, broader national strategies emphasize the role of cultural confidence and artistic engagement in promoting holistic human development and mental well-being (Ministry of Education of the People’s Republic of China, 2025). Our findings provide robust empirical support for these policy directions. The sequential mediating effect discovered in our study underscores that educational interventions should not merely mandate superficial or behavioral participation in art activities. Instead, educators and mental health practitioners should focus on cultivating students’ aesthetic perception and awakening their internal aesthetic needs. By fostering environments that encourage openness to experience and deep cognitive engagement with the arts, universities can help students internalize aesthetics as a fundamental psychological need. Once this need is established and fulfilled, it serves as a sustainable, intrinsic source of both subjective and psychological well-being, aligning perfectly with the contemporary educational goal of fostering psychologically resilient and culturally enriched youth.

Consequently, future intervention programs could focus on enhancing college students’ art-related aesthetic perception and needs as an indirect means of improving their well-being. In addition, longitudinal studies are recommended to further establish causal relationships.

## 6 Conclusion

In sum, within the context of Chinese young adults, this model illustrates how openness to experience is related to well-being through a “perception–need–fulfillment” pathway. While art-related aesthetic perception is rooted in cognition, it becomes impactful when translated into art-related aesthetic needs and subsequently fulfilled through meaningful engagement with the arts. This process underscores the psychological significance of aesthetic experience and highlights the role of openness to experience as a personality trait that indirectly enhances well-being through the activation and satisfaction of aesthetic motivations.

## Data Availability

The raw data supporting the conclusions of this article will be made available by the authors, without undue reservation.
