# Supplementary material for: The relationship between openness to experience and college students’ well-being: the mediating roles of art-related aesthetic perception and needs
Source: Front Psychol. 2026 Jun 15;17:1834660. doi: 10.3389/fpsyg.2026.1834660 (PMC13311017; doi:10.3389/fpsyg.2026.1834660)
Supplement: Supplementary file 1 [file Data_Sheet_1.PDF]

Supplementary Material

The model demonstrated a good fit: χ^2/df = 3.347, *CFI* = .971, *TLI* = .957, *GFI* = .955, *RMSEA* = .065, *SRMR* = .042. The significance of the indirect effect was tested using the bootstrap method (with 5000 resamples), and the results showed the following: (1) Openness to experience had no significant direct effect on well-being (β = .02, *p* = .424 > .05, *95% CI* = [-.086, .134]) ; (2) Openness to experience had a significant indirect effect on well-being (β = .08, *p* = .03< 0.05, *95% CI* = [.017, .140]) through art-related aesthetic needs; (3) Openness to experience had a significant indirect effect on well-being (β = .17, *p* < .001, *95% CI* = [.109, .238]) via art related aesthetic perception and needs. Overall, openness to experience did not directly affect well-being among university students, but rather influenced both through art-related aesthetic perception and needs. Detailed results are presented in Figure 3.

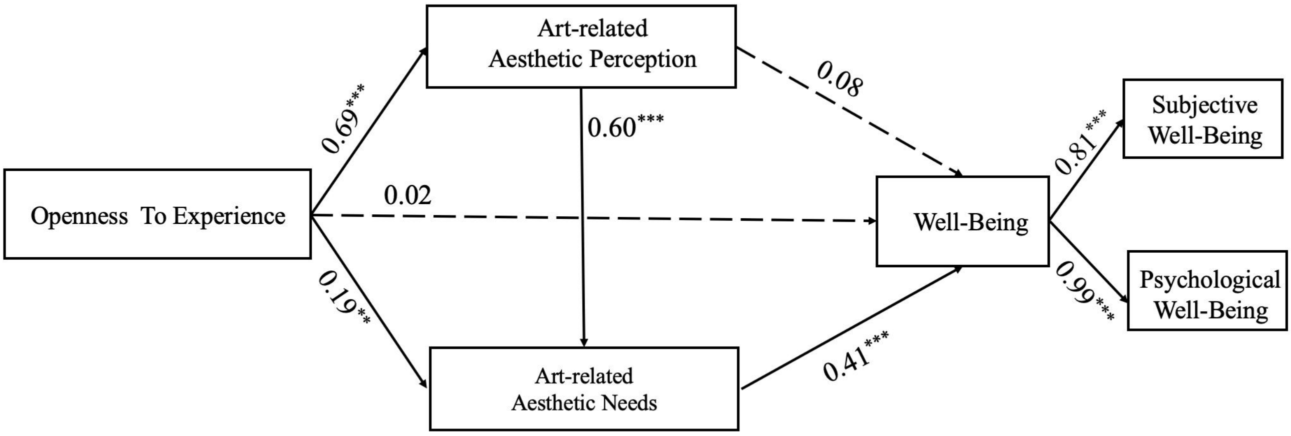

Fig.3. Indirect Effect Model of Well-Being among University Students
Note1. $^{***}p$ < .001, $^{**}p$ < .01
